# Columba: an integrated database of proteins, structures, and annotations

**DOI:** 10.1186/1471-2105-6-81

**Published:** 2005-03-31

**Authors:** Silke Trißl, Kristian Rother, Heiko Müller, Thomas Steinke, Ina Koch, Robert Preissner, Cornelius Frömmel, Ulf Leser

**Affiliations:** 1 Institute of Informatics, Humboldt-Universität zu Berlin, Unter den Linden 6, 10099 Berlin, Germany; 2 Institute of Biochemistry, Charité Universitätsmedizin Berlin, Monbijoustraß e 2a, 10117 Berlin, Germany; 3 Zuse Institute Berlin, Takustrasse 7, 14195 Berlin, Germany; 4 Technische Fachhochschule Berlin, Seestr. 64, 13347 Berlin, Germany

## Abstract

**Background:**

Structural and functional research often requires the computation of sets of protein structures based on certain properties of the proteins, such as sequence features, fold classification, or functional annotation. Compiling such sets using current web resources is tedious because the necessary data are spread over many different databases. To facilitate this task, we have created COLUMBA, an integrated database of annotations of protein structures.

**Description:**

COLUMBA currently integrates twelve different databases, including PDB, KEGG, Swiss-Prot, CATH, SCOP, the Gene Ontology, and ENZYME. The database can be searched using either keyword search or data source-specific web forms. Users can thus quickly select and download PDB entries that, for instance, participate in a particular pathway, are classified as containing a certain CATH architecture, are annotated as having a certain molecular function in the Gene Ontology, and whose structures have a resolution under a defined threshold. The results of queries are provided in both machine-readable extensible markup language and human-readable format. The structures themselves can be viewed interactively on the web.

**Conclusion:**

The COLUMBA database facilitates the creation of protein structure data sets for many structure-based studies. It allows to combine queries on a number of structure-related databases not covered by other projects at present. Thus, information on both many and few protein structures can be used efficiently. The web interface for COLUMBA is available at .

## Background

Biological databases have become a major resource for researchers in life science. With the constantly increasing number of data deposited and the computational tools evolving, the focus of research has shifted from the study of a single gene towards an intra- and inter-species comparison of genes and gene products. This trend can also be seen in the field of structural biology, where the number of protein structures deposited in the Protein Data Bank, PDB [1] is increasing rapidly. However, looking at the structure alone is not sufficient for a comprehensive study of the various types of relationships between proteins. Other types of information, such as functional and structural annotations of proteins, also have to be taken into account.

Oberg and colleagues [2] compared the results from infrared and circular dichroism spectroscopy with the actual 3D structure of a protein to gain insight into the relationship between assigned protein secondary structures and spectral band shape. To carry out this study, they had to compile a set of proteins based on the folding classification as defined by CATH, the content of secondary structure elements computed by the DSSP program, and the commercial availability of the proteins. Martin and colleagues [3] systematically explored the relationship between the folding classification from CATH and the classification of proteins into ENZYME classes. For that purpose, they needed groups of structurally resolved proteins belonging to one of the six main ENZYME classes. In both examples, the first step in the experiments was the compilation of a set of protein structures based on the structure itself and on folding classification, sequence properties, enzymatic activity, and other types of information.

Researchers have several possibilities to collect information on protein structures. First, entries in the PDB itself contain a set of full text information and often are annotated with links to external data sources. However, PDB entries are not curated, only archived by the PDB team. This has two consequences. First, the data are not constantly updated and therefore quickly become out-of-date. Second, the annotation provided by different submitters is highly heterogeneous and does not follow a standardized nomenclature. As a consequence, searching the PDB for annotations is an error-prone task. Annotations may be incomplete or inconsistent with standard nomenclature, spelling errors and uncontrolled usage of abbreviations prevent an efficient textual search, and literature references or links to functional and structural databases may be outdated or missing. Examples of such problems are described in [4]. This lack has led to a number of second-party databases that digest PDB entries and attach a wealth of links to relevant databases. The two best-known sources of that kind are probably PDBsum [5] and the IMB Jena Image Library [6]. Both store hyperlinks to external databases and not the actual information. Therefore they are well suited for human browsing of single entries, but inadequate for working with sets of structures and their properties. Imagine a researcher wants to compile the set of DNA binding proteins from mammals resolved by X-ray crystallography with a resolution lower than 3.2 Å. Solving this task can be achieved by using either PDBsum or the IMB databases, but it requires extensive manual work or the coding of specialized scripts [7].

To overcome this problem, we created COLUMBA, a database of information on protein structures that physically integrates information from twelve protein structure related data sources into a single data warehouse. Besides the protein structures themselves, COLUMBA covers structural and sequence-based classification schemes, functional annotation, secondary structure elements, and participation in metabolic pathways. Links between these data and the protein structures, both on the chain, compound, and entry level, are either taken from the second-party databases or are computed inside COLUMBA, leading to more accurate and more current information than available in the PDB itself and as current as possible, we compute links between chains and Swiss-Prot entries based on sequence similarity, thus cross-referencing 68% of the PDB entries to a Swiss-Prot sequence.

## Construction and content

### Data sources

COLUMBA is centered around PDB entries [1]. For each entry we store general information like the experimental method, resolution, deposition date, and author. Each PDB entry is organized in compounds, which represent biological units, and each compound has one or more chains. A compound, for which an enzyme classification (E.C.) number exists, is annotated with information from ENZYME [8] for the enzyme name and biochemical reaction, and with data from the Kyoto Encyclopedia of Genes and Genomes, KEGG [9] for the participation of that enzyme in metabolic pathways. COLUMBA also integrates data from the Roche Biochemical Pathway Map [10].

To gain information about protein domains, entries from the protein-fold classification databases SCOP [11] and CATH [12] are linked to protein chains. Furthermore, each chain is assigned to a PISCES cluster [13]. PISCES groups protein chains according to their sequence identity and experimental properties into culled sets. For each chain, the secondary structure is computed using the DSSP program [14]. Links to Swiss-Prot entries [15] were retrieved from the PDBSprotEC database [16]. Exploiting the links from Swiss-Prot to other databases, PDB chains are connected to the NCBI Taxonomy database [17] and functional annotation from Gene Ontology [18].

### Architecture and database schema

**Figure 1:**
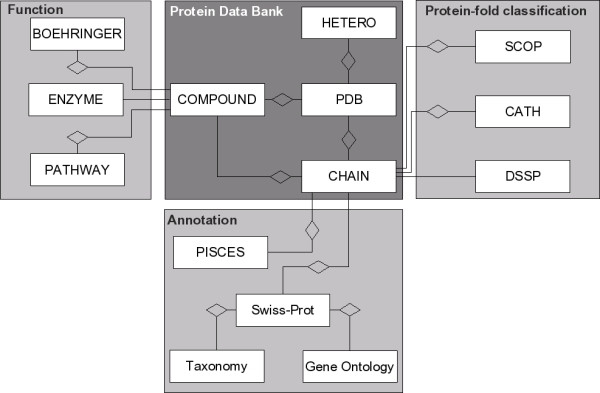
**Schematic entity-relationship model of COLUMBA**. The dark gray part in the middle is the subschema that originates from the Protein Data Bank (PDB). The other subschemas are represented by a single box indicating the name of the data source and are grouped according to a broad classification of their content.

All data sources integrated into COLUMBA describe specific aspects of either PDB entries itself, their compounds, or their chains. We never mix data from different data sources with each other. This partitioning is directly reflected in the database schema (see Figure 1), where we model each data source as a different dimension in which protein structures are annotated. Each data source occupies its own, specialized subschema within the overall schema of COLUMBA. As an example, the subschema of KEGG consists of three tables, one for the metabolic pathway names, one for the enzyme names, and the third table stores information about enzymes participating in pathways. Each subschema is linked to the central subschema representing PDB entries. This "separation of concerns" is also reflected in the Web interface.

### Integration of data sources into COLUMBA

COLUMBA is implemented on top of the open source database system PostGreSQL [19]. It currently integrates data from the twelve data sources as shown in Table 1. The data from the original sources are available in different formats, such as flat files, database dump files, or pure HTML pages. We use parsers, written in Python and Perl, respectively, to populate COLUMBA with the data obtained in non-relational representation. For PDB we use our own parser derived from the BioPython project [20]. To upload Swiss-Prot, Gene Ontology, and NCBI Taxonomy we use the parsers and schema provided in the BioSQL project [21]. After parsing each data source into a separate database schema, the data in those schemas are mapped into the COLUMBA target schema. Program source of our parsers is available on request. The connections between data sources and the PDB data are generally established by using existing links. Links from PDB to ENZYME, KEGG, and the Boehringer map are obtained through the E.C. number given in PDB entries. DSSP secondary structures are computed directly on the chains. The connection between PDB chains and Swiss-Prot entries is established by using the information from the PDBSprotEC database [16]. Swiss-Prot is also used as intermediate information for connecting PDB entries to the NCBI Taxonomy and Gene Ontology Annotation [22].

### Annotation workflow

The annotation workflow populates the COLUMBA data warehouse and establishes connections between PDB entries and the other data sources. Each data source is represented by a software module implementing a fixed interface. Once a new PDB entry is written into COLUMBA, a workflow manager triggers each module, which adds annotations to the entry. The implementation of modules varies according to the nature of the data source. For instance, the DSSP module calls the DSSP program to compute the secondary structure for each chain, whereas the SCOP module searches PDB and chain identifiers in external files. Our annotation pipeline is able to handle logical dependencies between different modules. This architecture allows to include a new data source by just extending the database schema for the new tables, and implementing an appropriate module.

### Content of COLUMBA

COLUMBA is populated with data using the annotation workflow described in the previous section. New entries from the Protein Data Bank are added regularly to COLUMBA, and links to the other data sources are established upon this import. Data sources with a release policy, such as Swiss-Prot, SCOP or CATH are updated according to new releases. All other data sources are updated as new data becomes available. Table 2 lists the number of PDB entries, broken down to compounds and chains that have an annotation in the respective sources and combinations of sources.

## Utility

COLUMBA is a relational, integrated database of information on protein structures and is specially designed to support the creation of sets of protein structures sharing annotations in any of the data sources. Sets as those described in the introduction can be compiled with a few mouse-clicks using COLUMBA.

### Web interface

COLUMBA can be searched through a web interface available at . The interface allows two types of queries: Full text search as well as data source and attribute specific searches. In both cases, the query results in a list of PDB entries with their corresponding chains.

**Figure 2:**
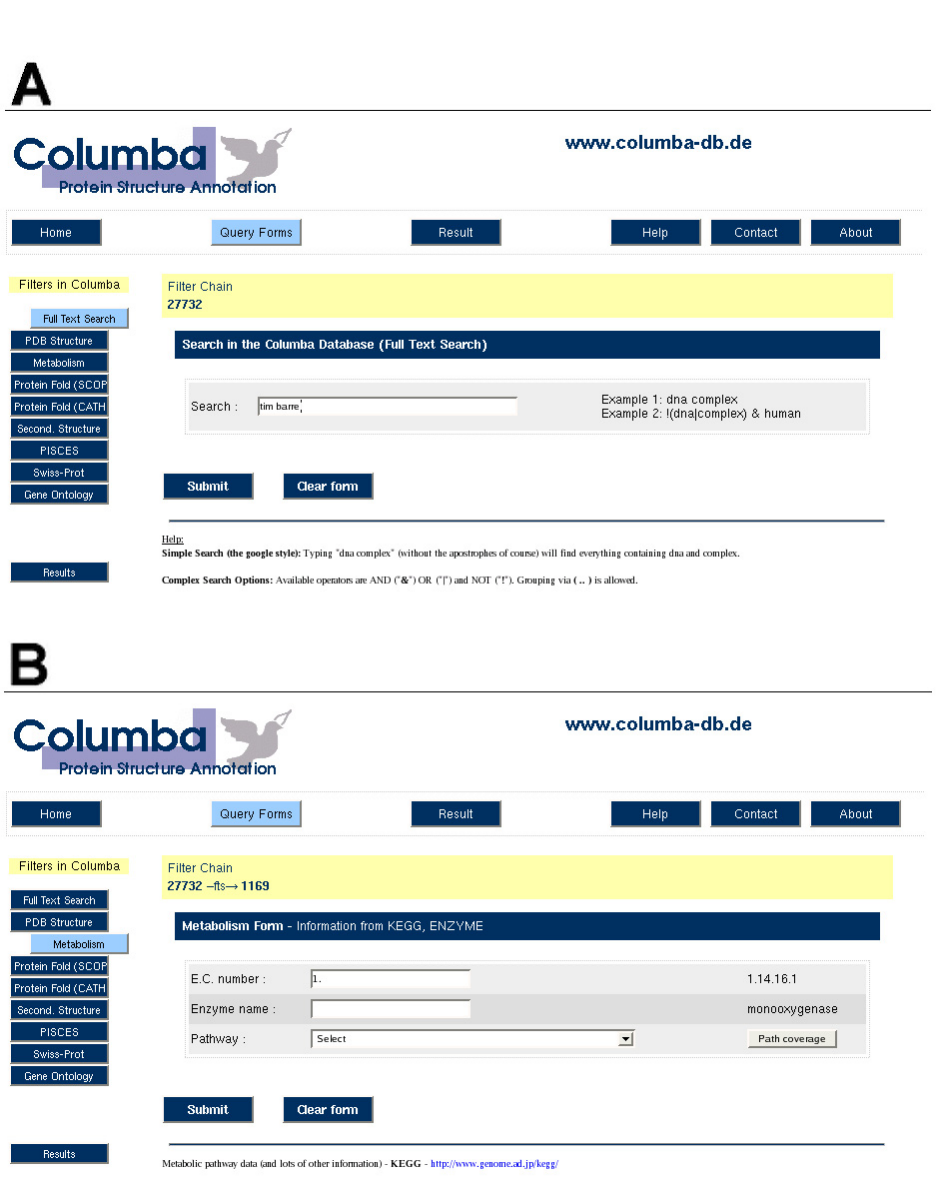
**Screen shots of COLUMBA web-forms. **(A) Interface for the full text search. (B) Query form for the metabolism information, where the result set can be restricted by information from ENZYME and KEGG.

For convenience and as a quick-start, COLUMBA can be searched by using a standard keyword search over all textual fields in COLUMBA (Figure 2A), including the annotation given by the PDB, enzymatic, metabolic, taxonomic, and the protein-fold classification information. Keywords can be combined using logical AND, OR, and NOT operators. The keyword search performs a simultaneous request over the content of all integrated data sources, and is thus a quick and easy-to-use option for finding interesting protein structures. However, it does not allow for source- or attribute specific queries, e.g., for finding all protein structures, which are specifically annotated in CATH as containing a Rossmann fold. The main focus of COLUMBA is the compilation of sets of structures sharing properties from different second-party databases. To support such queries, we have created a specialized web interface based on the paradigm of query refinement. This process is best understood as having an initial data set, which is subsequently reduced by applying different filters. In our case, the initial data set contains the entire set of PDB entries. For each of the data sources integrated into COLUMBA, the user may specify source-specific filter conditions using a proper web form (see Figure 2B). The source specific forms can be found by using the labeled buttons on the left side of the web page. After entering conditions in a form, those PDB entries that do not fulfill the stated conditions are removed from the current set of results. Several forms can be used consecutively, thus restricting the original set of all PDB entries by conditions on multiple data sources. Conditions on different sources are always logically connected by an AND. The available search operators depend on the specific field and data source, ranging from numerical comparisons to substring matching and traversal of ontological structures. To guide the user, COLUMBA constantly shows the current number of qualifying PDB entries after each query step in the header of the page. This demonstrates the consequences of adding, deleting, or changing conditions and helps to prevent the over-specification of search conditions leading to empty sets. Note that the full-text search can be used as an additional restriction condition on the result set, which has turned out to be a quite powerful feature of the search interface.

**Figure 3:**
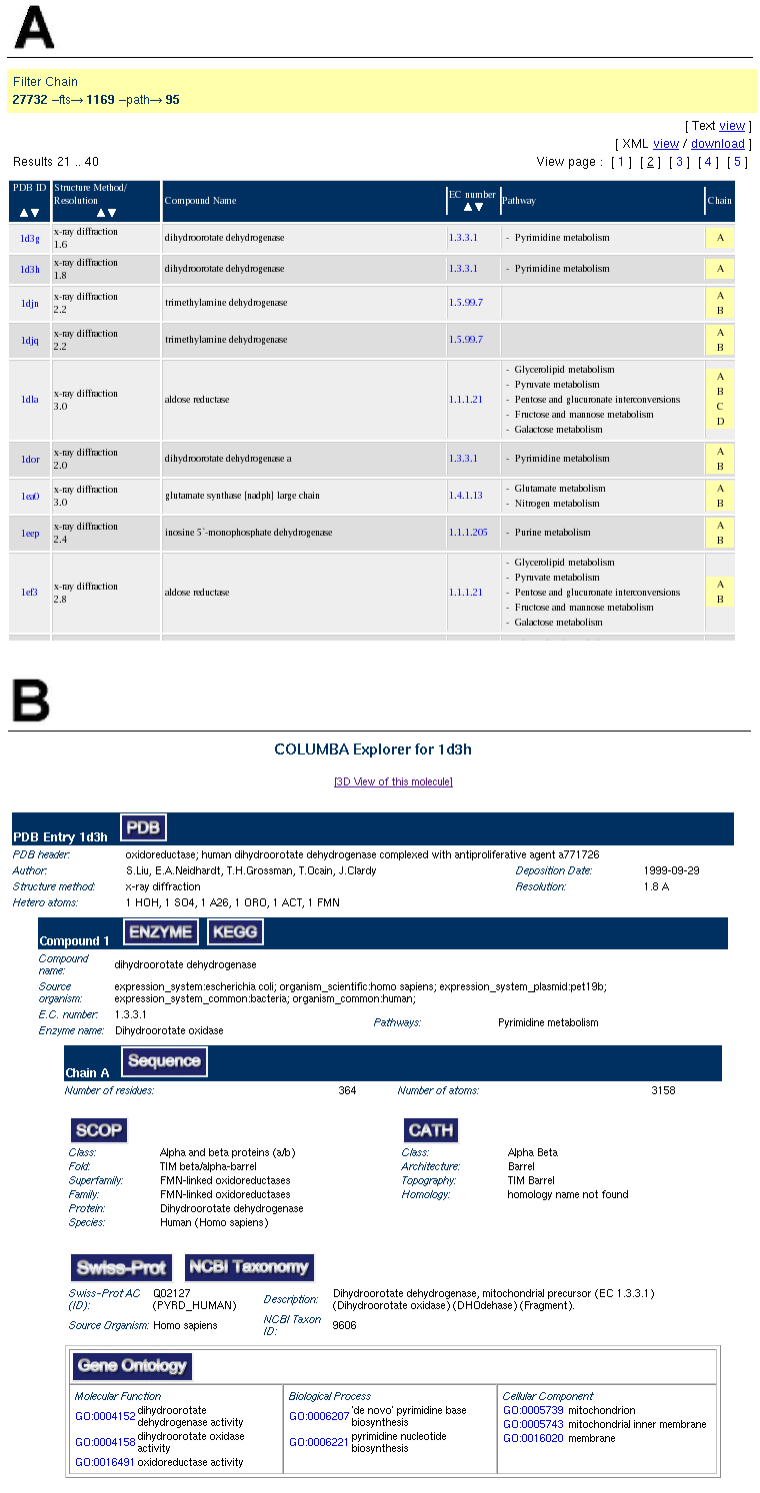
**Screen shots of COLUMBA query results. **(A) Result set for a query requesting structures from the ENZYME class '1.-.-.-' combined with a full text condition on 'TIM barrel'. (B) COLUMBA Explorer detailed view of the PDB structure 1d3h.

Once the user has specified all desired conditions, COLUMBA computes the qualifying set of protein structures. This list of results (see Figure 3A) gives basic information, such as PDB ID, experimental method, compound name, and chains for each entry. The PDB entry ID links to the COLUMBA Explorer view for that particular entry. The Explorer (see Figure 3B) shows all information stored in COLUMBA for that PDB entry. This includes the experimental method and resolution for each entry and compound name, metabolic information, and the source organism for each compound. Detailed information is given for each chain, including protein-fold classification from SCOP and CATH, data from the according Swiss-Prot entry, Gene Ontology annotation, and NCBI taxon name. These data can also be viewed or downloaded in XML format. We also provide on-line molecular visualisation via JMol [23], and links to the original data items in the respective databases.

To further enhance the search capabilities of the web interface, it is possible to upload a file containing a set of PDB identifiers. Thus, a user can view all data in COLUMBA for the entries in his list and create subsets of protein structures from the list by entering conditions on second-party annotations. Thereby, the COLUMBA web interface greatly reduces the required time to collect additional information for entries in any list of PDB entries.

### Example of use

Consider a query for all compounds from ENZYME class '1.-.-.-' containing a chain with a TIM barrel fold (see Figure 3A). To compute this set, a user first specifies 'TIM barrel' in the full text search form, which returns all PDB chains with the keyword 'TIM barrel' in any of the data sources, including the PDB, SCOP, and CATH annotation. Next, the set of all proteins fulfilling this condition can be intersected with the result of the search for the ENZYME class in the metabolism form. The intersection contains 95 PDB structures. However, using the full text search is only one option for finding the appropriate answer. In general, different answers are possible for a given question depending on the preferences and trust of the user in different databases. Consider again the example given above. If a user has high confidence in either CATH or SCOP, he may specify a condition using the CATH or SCOP form, respectively, instead of performing the full text search for 'TIM barrel'. This results in 79 entries when relying only on CATH and 90 entries for SCOP. The user might even want to restrict the search to only those chains that are annotated as containing a 'TIM barrel' fold in both CATH and SCOP. The returned set has 79 PDB protein structures. These differences result from the fact that COLUMBA usually only takes the cross-references given in the original data and does not curate or amend the content of the integrated databases.

### Applications of COLUMBA

The web interface is designed to compile sets of protein structures sharing properties from protein structure related sources, but it is possible to tackle more sophisticated issues by exploiting the relational data warehouse of COLUMBA. We show a number of applications where we used SQL (Structured Query Language) to retrieve information.

A research question concerning the participation of enzymes in metabolic pathways arose from an article from Martin et al. [3] that investigated the relationship between the protein classification of ENZYME and the folding classification of CATH. One finding at that time was that the known enzymes in the glycolytic pathway contained a very limited set of different CATH architectures and topologies. This naturally raises the questions whether this is the case for other metabolic pathways as well. We used COLUMBA to address this problem, combining PDB data, information on metabolic pathways from KEGG, and the CATH classification.

Each KEGG pathway consists of a number of enzymes related to a PDB compound. Those compounds are linked to the respective chains, which in turn are cross-referenced to CATH classes, architectures, and topologies. We computed the number of occurrences of CATH classes for the set of enzymes in a pathway containing more than 10 enzymes and having a coverage of at least 50% with PDB structures that are also annotated in CATH. For all qualifying pathways the figures are given in Table 3.

The first striking fact is that only 26% of the enzymes participating in KEGG pathways do have annotated chains in CATH. This is because just 34% of the enzymes in KEGG are structurally resolved, of which several are not annotated by CATH. The enzymes within the annotated set contain four times as many domains with an Alpha/Beta class in CATH than Mainly Alpha and Mainly Beta, respectively. In comparison, for all proteins annotated by CATH the Alpha/Beta class only occurs twice as often as each of the other two folds.

**Figure 4:**
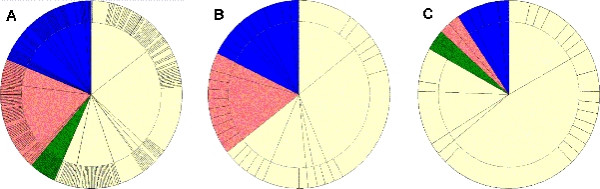
**The CATH wheel for KEGG pathways**. The color of the CATH wheel represents the CATH classes, where yellow stands for alpha/beta, red for mainly alpha, blue for mainly beta, and green for Few Secondary Structures. The inner circle represents the CATH architectures (C.A.), where the width of each segment represents the number of enzymes found to exhibit that architecture. The outer circle stands for the Topology (C.A.T.). (A) shows the distribution of all enzymes participating in KEGG pathways with the '3-layer(aba) sandwich' representing the largest architecture. (B) shows the CATH wheel for the pathway 'Pyrimidine metabolism' while (C) for 'Glycolysis/Gluconeogenesis'.

In Figure 4 the subdivision of all enzymes (Figure 4A) as well as of selected pathways into classes, architectures, and topologies from the protein-fold classification CATH is shown in 'CATH wheels'. The predominant CATH architecture in all three 'CATH-wheels' is the 3-Layer(aba) Sandwich, with the 'Rossmann fold' comprising the biggest topology. In Figure 4B the Pyrimidine metabolism is shown. As we can see, the shares of the different classes are almost equal to the distribution of classes in all enzymes. Figure 4C shows the Glycolysis/Gluconeogenesis pathway, where in 1998 only 11 enzymes were known. These enzymes exhibited mostly an Alpha/Beta fold. By now, 24 enzymes are resolved and structurally classified by CATH, which lead to more domains that differ from the predominant Alpha/Beta fold. As more and more enzymes become structurally resolved in the future, this picture will shift yet again.

## Discussion

### Related work

The most frequent approach to the interconnection of data on protein structures that are spread over multiple original data sources is the usage of hyperlinks. Examples are PDBsum [5] and the IMB Jena Image Library [6]. This method is well suited for human browsing of single entries, but as soon as it comes to handling sets of objects, following many hyperlinks becomes a tedious and time consuming task. Efficient handling of sets can only be achieved if data are physically integrated into a single system. In the protein structure world, there are three main such databases apart from COLUMBA. 3DinSight [24] focuses on visualization of sequence features such as PROSITE patterns or altered positions in the 3D structure. iProClass [25] concentrates on protein sequence and integrates 50 different databases using so-called 'rich links'. Finally, BioMolQuest [26] integrates in total four data sources, thus storing only a subset of the information available in COLUMBA. Currently, the Protein Data Bank itself is preparing a new web interface to provide not only the links to related sources, but the actual information from SCOP, CATH, and the Gene Ontology. These are only a subset of the sources integrated in COLUMBA. COLUMBA's functionality could also have been achieved by implementing specific modules for SRS. However, we early on decided to use relational database technology instead of the highly proprietary SRS languages and methods.

Two groups currently address the problem of inconsistent use of terminology in the PDB: the PDB uniformity project [4] and the Macromolecular Structure Database MSD [27]. Both projects aim at correcting PDB entries, unifying terminology, and adding or updating links to scientific references. The MSD also addresses the linkage of PDB chains to Swiss-Prot entries. We hope that these efforts will make our work easier in the near future, for instance if the PDB entries themselves come with consistent and structured taxonomic information.

COLUMBA currently integrates twelve data sources concerned with different aspects of protein sequences and structures. Notably, COLUMBA does not store the coordinates of structures themselves but is designed to enable users to find 'the right' set of structures based on annotations. This is by intention, since there are already many programs that can efficiently parse, visualize, or compare protein structures from PDB files.

An important design principle of COLUMBA is that it never mixes data from different sources into a single table. Each data source is considered as a dimension in which PDB entries, compounds, and chains are annotated. We call this approach multidimensional data integration [28], which is inspired by data warehouse design, where facts, e.g., sales, are described by dimensions, such as store, product, or customer [29]. The resulting database schema is called star schema in correspondence with the visual appearance. We also use a star schema like structure with the tables holding information from protein structures in the center of a set of tables containing the data from other data sources.

Our approach is in contrast to projects that aim at a tighter semantic integration, merging logically similar types of information into a single table. Such a semantic integration approach was for instance followed in the TAMBIS project [30]. However, we strongly believe that merging data from different databases is counterproductive for the biologist because it blurs important differences. On the other hand, keeping data separated inevitably leads to a certain degree of semantic redundancy, i.e., different schema elements provide the same type of information. For instance, functional annotation of proteins is encoded both in Swiss-Prot keywords and Gene Ontology terms; 'TIM barrels' are annotated in CATH, SCOP, and the PDB annotation itself. But this redundancy does not originate from data duplication, but rather from evidence obtained independently by different people or by different experiments. These evidences are important in their own right.

We believe that the advantages of our approach prevail for mainly two reasons:

• Users recognize the origin of the data they query and obtain as result. In our experience, biologists often have their favorite set of databases, where they know about the pitfalls and peculiarities. By keeping data separated, personal preferences or differences in trust in particular databases can be expressed and the results can be judged based on prior experience.

• Subtle differences in the semantics of fields of different databases are conserved. For instance, both Swiss-Prot keywords and GO annotations express functional annotation. However, the process of creating this annotation is quite different, and it is often meaningful to discriminate between the two.

Furthermore, separating data and software for the different data sources greatly simplifies system maintenance. Changes to data sources, including the deletion or addition of data sources, only affect a well defined part of the schema and of the web interface.

Our perception of considering annotation sources as dimensions describing some primary objects is also followed in the EnsMart project [31]. EnsMart uses a 'reversed star schema' to connect genes with different types of information, such as genomic position, transcription factors, or expression data. The data are queried through a generic web interface, which also allows source-specific queries and their combinations. Conceptually, EnsMart and COLUMBA are very similar, but they work on totally different types of data. Moreover, COLUMBA is directly designed for handling annotations of protein structures, which has advantages in terms of result visualization and search options.

## Conclusion

COLUMBA has proven to be very useful for a number of tasks in our own structural research. Generating sets of structures, which previously required days of manual browsing or writing of parsers, now only takes a few mouse clicks, or an SQL query. Once the set of PDB entries and chains is obtained, there are many other programs for visualizing or comparing structures. COLUMBA's future development will further concentrate on annotation of structure in contrast to the structure and its coordinates itself. The next data sources to be integrated are those covering protein domains and motifs, i.e., InterPro [32] and its relatives. In the long run, we will push COLUMBA towards a medical orientation. Obvious candidates for being integrated are literature abstracts from Medline and the OMIM database [33]. The LIGAND database [34] will provide information about small molecules interacting with proteins to use COLUBMA for the prediction of drug target sides. Moving towards medical data is a natural next step since much of structural research, including our own [35] is concerned with drug development.

## Availability

The database is available at .

## Authors' contributions

ST designed and implemented the web-interface. KR was responsible for the implementation of the annotation workflow. HM helped importing PDB data. UL designed the overall architecture and co-supervises the project. TS maintains the server infrastructure. IK provided data on the classification of protein folds, and RP on protein ligands. CF co-supervised the project and was the main source of motivation for building an integrated protein annotation database.

## Figures and Tables

**Table 1:** Data sources integrated in COLUMBA.

Source	download page	format	Parsed by
PDB		flat file	BioPython
SCOP		flat file	BioPython
CATH		flat file	own
DSSP	computed	-	own
ENYZME		flat file	BioPython
Boehringer		HTML	own
KEGG		HTML	own
Swiss-Prot		flat file	bioSQL
GO		flat file	bioSQL
GOA		DB dump	COPY
Taxonomy		flat file	bioSQL
PISCES		DB dump	own

The forth column gives the parsers used.

**Table 2:** Number of entries from the PDB.

from PDB	to	Number of entries
	SCOP	42 908
	CATH	32 825
	DSSP	54 028
chains (total 60 241)	Swiss- Prot	36 651
	NCBI Taxonomy	36 651
	Gene Ontology via GOA	36 008
	PISCES	8 367
	SCOP & CATH	32 439
	SCOP & CATH & Swiss-Prot	27 972

	Enzyme	12 510
	Boehringer	5 029
7162 compounds (total 33 779)	KEGG	7 162 7 162 9 172
	Enzyme & KEGG	7 162
	Enzyme & SCOP & CATH	9 172
	Enzyme & SCOP & CATH & KEGG	5 054
	Enzyme & Swiss-Prot	9 440

entries (total 26 104)	all minus PISCES	2 868
	all	621

They are divided into compounds and chains, which link to second-party databases and selected combinations of them.

**Table 3:** The number of enzymes for selected metabolic pathways from KEGG.

Metabolic pathway	Enzyme total			CATH class			
	Total	with str.	coverage	a / b	a	b	Few
all pathways	1 952	508	26,0	443	114	107	15
Fatty acid biosynthesis (path 1)	14	7	50,0	6	0	2	0
Oxidative phosphorylation	10	5	50,0	3	3	3	1
Streptomycin biosynthesis	14	7	50,0	6	0	1	0
Pyrimidine metabolism	59	30	50,8	29	6	5	0
Selenoamino acid metabolism	21	11	52,3	11	2	2	1
Pentose phosphate pathway	33	18	54,5	17	2	2	2
Methionine metabolism	23	13	56,5	13	3	1	1
One carbon pool by folate	23	13	56,5	13	2	1	0
Phe, Tyr and Trp biosynthesis	31	19	61,2	18	6	2	0
Glycolysis / Gluconeogenesis	38	24	63,1	24	2	5	2
Reductive carboxylate cycle (CO2 fixation)	13	9	69,2	8	3	1	1
Aminoacyl-tRNA biosynthesis	21	16	76,1	16	8	6	0
Carbon fixation	23	18	78,2	18	2	3	0

The sum of observations in CATH classes can be higher than the number of enzymes with structures from the pathway, because in one chain, several domains with distinct folds can occur.
